# Reliability of Three-Dimensional Pseudo-Continuous Arterial Spin Labeling MR Imaging for Measuring Visual Cortex Perfusion on Two 3T Scanners

**DOI:** 10.1371/journal.pone.0079471

**Published:** 2013-11-20

**Authors:** Diandian Huang, Bing Wu, Kaining Shi, Lin Ma, Youquan Cai, Xin Lou

**Affiliations:** 1 Department of Radiology, PLA General Hospital, Beijing, China; 2 Department of Radiology, Beijing Military General Hospital, Beijing, China; 3 MR Research (China), General Electric Company GE (China) Co., Ltd.-Healthcare, Beijing, China; University Medical Center (UMC) Utrecht, The Netherlands

## Abstract

Cerebral blood flow (CBF) in the human primary visual cortex is correlated with the loss of visual function in neuro-ophthalmological diseases. Advanced three-dimensional pseudo-continuous arterial spin labeling (3D pCASL), as a non-invasive method to access the CBF, can be a novel measurement to detect the visual cortex. The objective of the study was to assess the intra- and inter-scanner reliability of 3D pCASL of the visual cortex in healthy adults and suggest the selection of different post-labeling delay times (PLDs). For this reason, 3D pCASL was conducted in two 3.0T MR three times with twelve healthy volunteers at an interval of 10–15 days. The 1st and 3rd tests were performed on scanner-1, and the 2nd test was performed on scanner-2. The value of the CBF was abstracted from the visual cortex with two PLDs. The intra- and inter-scanner reliability and reproducibility were evaluated with the intraclass correlation coefficient (ICC) and Bland-Altman plots. By estimating the mean value of the CBF in the visual cortex, the intra-scanner results demonstrated the higher reliability (ICC for PLD = 1.5 second presented at 0.743 compared with 0.829 for PLD = 2.5 seconds), and the Bland-Altman plots showed the reproducibility at a longer PLD. We conclude that the calibrated 3D pCASL approach provides a highly reproducible measurement of the CBF of the visual cortex that can serve as a useful quantitative probe for research conducted at multiple centers and for the long-term observation of the clinical effects of neuro-opthalmological diseases.

## Introduction

Differences in the cerebral blood flow (CBF) of various visual cortices were correlated with differences in visual function in glaucoma [1]. Glaucoma patients often suffer silent vascular insults from cerebral small blood vessel disease. On the one hand, small blood vessel vasculopathy predisposes towards decreased compensatory responses to extreme perfusion fluctuations [2]. On the other hand, small blood vessel disease is typically silent, and some ischemic lesions in watershed areas such as the visual cortex may injure the posterior visual pathways without being clinically obvious [2]. These results confirmed the importance of vascular factors in the development of glaucomatous neuropathy.

Many studies have variable access to monitor glaucomatous CBF changes in humans. Compared with the nuclear medical technology, arterial spin labeling (ASL) in magnetic resonance (MR) imaging can provide a non-radiative and noninvasive CBF measurement in a more convenient way. Two-dimensional pulse ASL has been proven to be effective in the investigation of visual function-related perfusion disparities [3]. As a novel developed ASL technique, three-dimensional pseudo-continuous arterial spin labeling (3D pCASL) has a better signal-to-noise ratio (SNR), more uniform perfusion effect and better immunity to the transient time [4], [5], whereas the fast spin-echo (FSE) spiral acquisition approach exhibits less ghosting and signal loss.

The 3D pCASL, which in response to cerebral functional activation, can be used to interpret the relationship between the CBF values of the visual cortex and visual dysfunction in human neuro-ophthalmological diseases such as glaucoma [1]. Compared with prior approaches [6], the 3D pCASL enables the measurement of cerebral perfusion without the demand for exogenous tracers, making it a promising technique for investigating perfusion in patients with renal failure and in patients who require repetitive follow-ups. There have been some studies that used non-invasive ASL technology to assess the reproducibility of the CBF [7], [8], [9], [10], [11]. Even so, such studies selected only one post-labeling delay time (PLD), the variation of which can lead to the underestimation of the CBF [12]. In addition, these studies closely followed the reproducibility of the whole brain, whereas this study focused on the visual cortex.

The current study was therefore driven by two main motivations: first, to quantify the between-subject reproducibility across the population and compare it with the single-subject reliability for 3D pCASL measurements and, second, to suggest the different PLDs in a systematic characterization of 3D pCASL relative to the CBF across human visual areas. By measuring these two sources of variance in collected data, we can begin to evaluate the application of the CBF to a population by quantifying the degree to which the variance in the population data can be attributed to measurement errors.

## Materials and Methods

### Ethics Statement

This study was approved by the Institutional Review Board of PLA General Hospital and the Institutional Review Board of Beijing Military General Hospital. Written informed consent was obtained from all participants.

### Participants

A total of fourteen healthy patients were screened, of one was excluded because of presence of claustrophobia during the test, and one because MRI with the necessary sequences of adequate quality was not available for analysis. There were twelve subjects were recruited in the study ultimately. Spontaneous fluctuations of CBF and the age influence on the CBF in healthy subjects have been proved [13], [14], the twelve healthy subjects (nine males and three females, between 27 to 48) have the three times scan same in the afternoon during 2pm to4pm at the mean interval of 13 days in two 3 Tesla MR imaging systems.

### Exclusion criteria for MR

The MR exclusion criteria included a history of claustrophobia, major psychiatric diagnoses, primary psychotic and affective disorders, and other types of neurodegenerative disease. Further criteria for exclusion included a history of cardiovascular disease, a history of stroke, the presence of two or more lacunar infarcts of 5 mm each, the use of anticoagulants, other known vasoactive medications that could affect the CBF and metal in the body that could not be removed. Due to the previous study [15], which found a significant global reduction with caffeine (20%) and tea (21%) in gray matter CBF, with no effect of decaffeinated tea, suggesting that only caffeine influences CBF acutely. The participants signed an agreement which implicated that caffeine or any beverages contained caffeine were prohibited and were inquired to confirm the attention before every test. No hypertension was observed in any subject at the time of the MR scans.

### Exclusion criteria for neuro-opthalmology

The neuro-opthalmological exclusion criteria included the following: glaucoma, non-glaucomatous secondary causes of elevated intraocular pressure (IOP) (e.g., iridocyclitis and, trauma), other intraocular eye diseases, other diseases affecting the visual field (e.g., pituitary lesions, demyelinating diseases, and diabetic retinopathy), medications known to affect visual field sensitivity, and problems other than glaucoma affecting color vision (as assessed by the Farnsworth D-15 color vision test). All the participants were required to keep their eyes closed during the test.

### MR Imaging and Data Processing

All volunteers were scanned three times on two MR GE 3.0T MR scanners (Discovery MR750) with the 3D pCASL fast spin echo (FSE) sequence using body coil transmission and 8-channel head coil reception. The two scanners were located at two different research centers. The first and third tests were conducted on scanner-1, and the second test was conducted on scanner-2. Different PLDs of 1.5 second (s) and 2.5 s were acquired during every scanning test. The time intervals between the tests ranged from 10 to 15 days. Before every scan, the root of the nose was located at the center of the magnetic body. The distance from the chin to the suprasternal notch and the distance between the top points of the auricular to the horizontal line from the top point of the superciliaris were controlled at the same level (62 mm) among the three tests.

Perfusion imaging was performed with pseudo-continuous labeling, background suppression, and a stack of spirals of 3D FSE imaging sequences. The 3D pCASL followed the method of Dai et al [16]. Selective inversion and saturation pulses were applied to a slab containing the imaged region and ending at the labeling plane. In addition to the background suppression pulses, inferior saturation pulses were applied to suppress the inflowing arterial blood spins after labeling was completed. A reference image volume for quantification was also acquired. The images were acquired with an interleaved 3D spiral fast spin echo sequence with the following parameters: 512 sampling points on eight spirals, spatial resolution = 3.64 mm, TR = 4590 (PLD = 1.5 s)/5285 msec (PLD = 2.5 s), TE = 10.5 msec, bandwidth = ±62.5 kHz, slice thickness = 4 mm, number of slices = 36, labeling duration = 1500 msec, acquisition time = 4:29 (PLD = 1.5 s)/5:09 (PLD = 2.5 s) minutes, field of view (FOV) = 24 cm, and number of excitations (NEX) = 3. The spiral fast spin echo sequence helped minimize the sensitivity to field non-uniformity.

In addition to the perfusion-weighted images, a high-resolution volumetric T1-weighted sequence (an inversion recovery [IR]-prepared fast spoiled gradient recalled echo [FSPGR] pulse sequence) anatomical images of the whole brain were acquired with the following parameters: TR = 8.2 msec, TE = 3.2 msec, bandwidth = ±31.2 kHz, TI = 450 msec, FOV = 24 cm, slice thickness  = 1 mm, number of slices = 156, acquisition time- = 4:08 minutes, matrix = 256×256, and NEX = 1.

The perfusion images were generated by pairwise subtraction between the control and tag pairs and were averaged over time. The quantification was performed using the previous model [17], with the inclusion of the finite labeling duration and correction for the incomplete recovery of the tissue signal in the reference image due to the saturation performed (t*_sat_*, 2,000 msec) before imaging [18]. The calculation of the flow was based on the following equation:
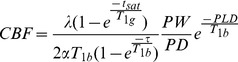
where T_1*b*_ is the T_1_ of the blood (1600 ms), T_1*g*_ is the T_1_ of the gray matter (1200 ms), α is the labeling efficiency (0.8), λ is the cortex–blood partition coefficient(0.9), t*_sat_* is the time of saturation performed before imaging (2,000 ms), τ is the labeling duration (1,500 ms) and PLD is the post-labeling delay time.

We used a gray matter T1 estimate of 1200 msec, an assumed blood T1 of 1600 msec, and a cortex–blood partition coefficient value of 0.9. The labeling efficiency was assumed to be 0.8 for the 3D pCASL.

The 3D IR-prepared T1-weighted images were used for image registration and normalization on a standardized space (Montreal Neurological Institute template, MNI space) within the Statistical Parametric Mapping (SPM8)(http://www.fil.ion.ucl.ac.uk/spm/software/spm8/) on MatLab 12b (MathWorks, Natick, MA). All quantitative CBF maps were normalized to the MNI space. Standard anatomical regions-of-interest (ROIs) from the WFU Pickatlas (Wake Forest University, http://fmri.wfubmc.edu/cms/software) were applied to the normalized images in SPM8. A predefined set of standard ROIs, including the Brodmann area (BA)17, BA18 and BA 19 (separated by the left and right and short for BA 17, BA 18 and BA 19), were used as templates for the ROI analysis. The standard ROIs of the reference image were generated using the Talairach Daemon database atlases [19], [20]. The classical cytoarchitectonic maps, including BA 17, BA 18 and BA 19 areas, which are based on a traditional tripartition of the visual cortex, were chosen [21], [22]. The mean CBF values were extracted for the inter- and intra-scanner comparisons.

The calculation of the SNR were followed up with the previous study [23].

### Statistical Analysis

The intra- and inter-scanner CBF values were mainly evaluated with the intraclass correlation coefficient (ICC) and presented by Bland-Altman plots. The ICC [24] measures the contribution of the between-subject variances to the total variance, typically ranging from 0–1. ICC values close to 1 indicate high reproducibility and reliability. For this research, two-way model average-measured ICC values were calculated using SPSS (v. 18.0, Chicago, IL). The data comparisons were presented using the method of Bland and Altman (Bland and Altman, 1996) on the website http://www-users.york.ac.uk/_mb55/meas/cv.htm. The Kolmogorov – Smirnov test was used to assess different value between the left and the right BA groups. All the variables were normally distributed. Levine's test was used to compare homogeneity of variance. The whole variables had homogeneous variance. Paired *t* tests were used to compare CBF changes between groups.

## Results

**Figure 1:**
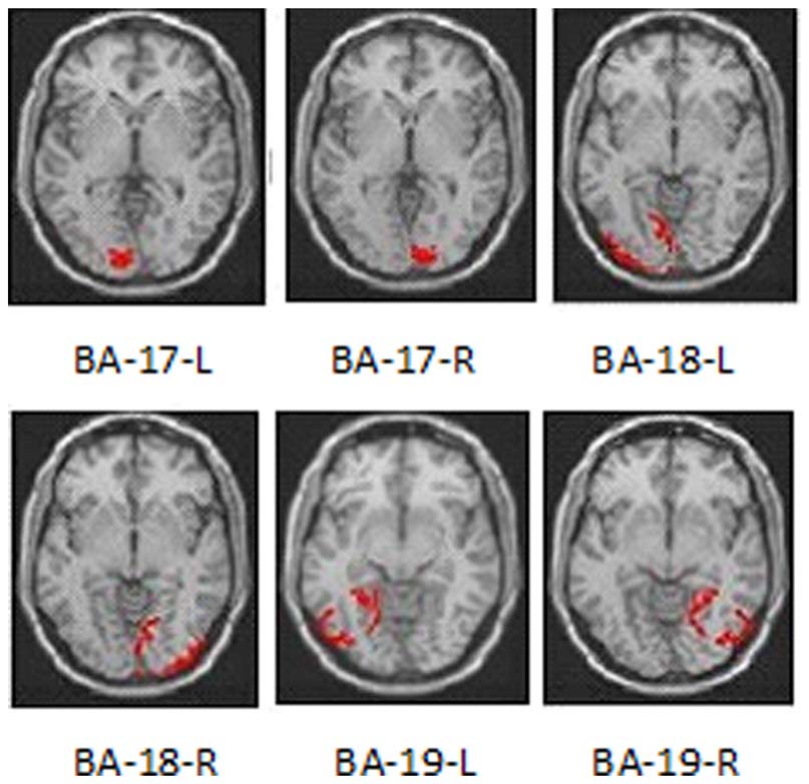
ROIs representation. ROIs included the Brodmann areas (BA) 17, 18, and 19 (divided into left and right). BA 17, 18 and 19 included the continuous layer while the largest one was selected as the representation.

**Figure 2:**
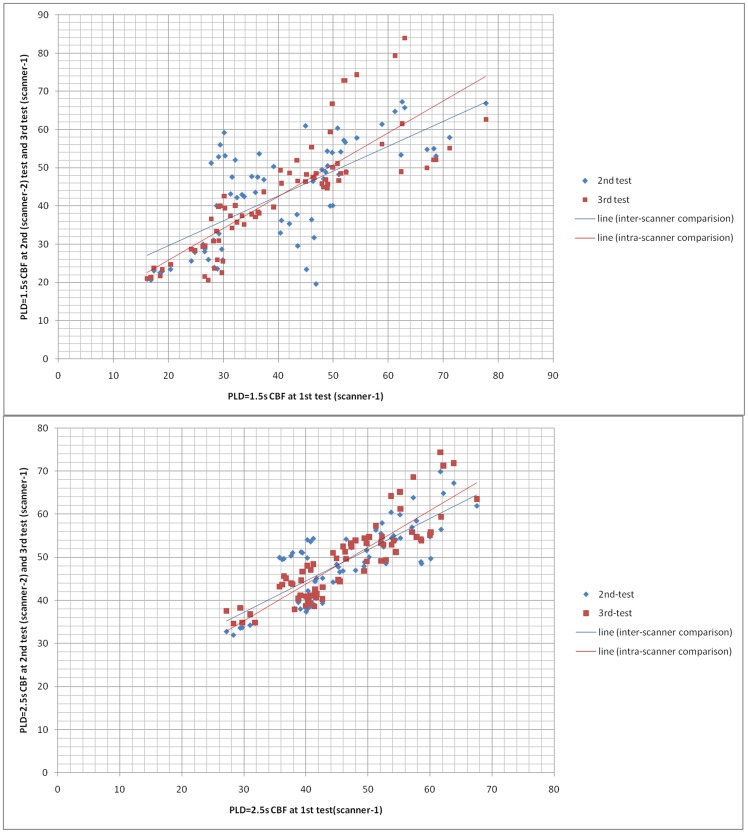
Correlation plots of the intra- and inter-scanner. Correlation plots for the test–retest CBF measurements from all ROIs in the twelve volunteers, derived from spatially normalized perfusion-weighted imaging data. The plots show that the correlations between the test and retest examinations were better with PLD = 2.5 s than with PLD = 1.5 s. Perfusion was measured in units of mL/100 cc gray matter/min.

**Figure 3:**
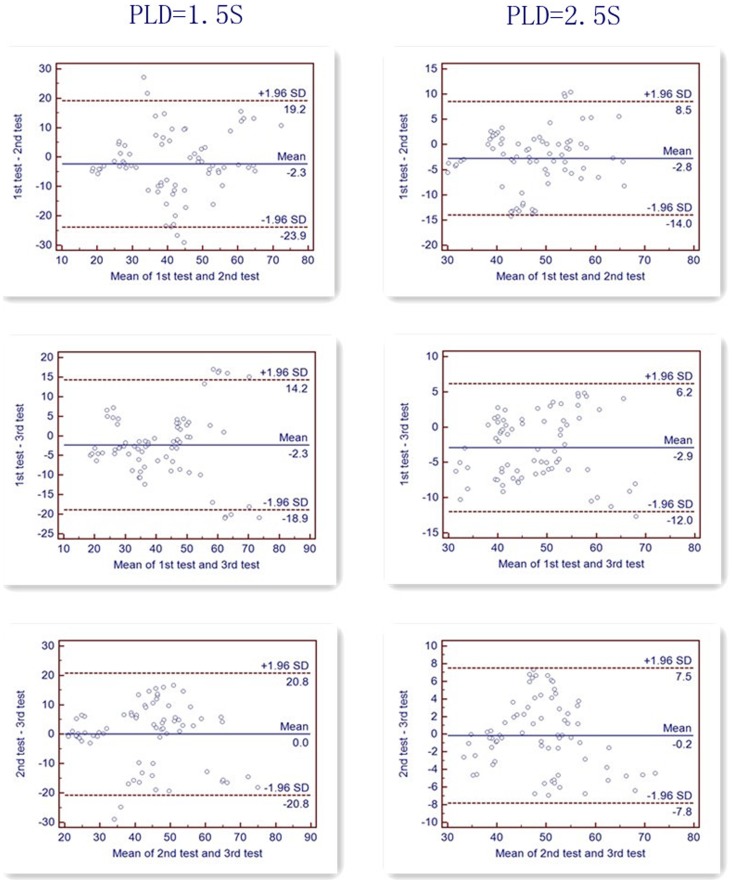
Bland–Altman plots together with the 95% CI. Bland–Altman plots of the CBF measurements from all ROIs in the twelve volunteers.

**Figure 4:**
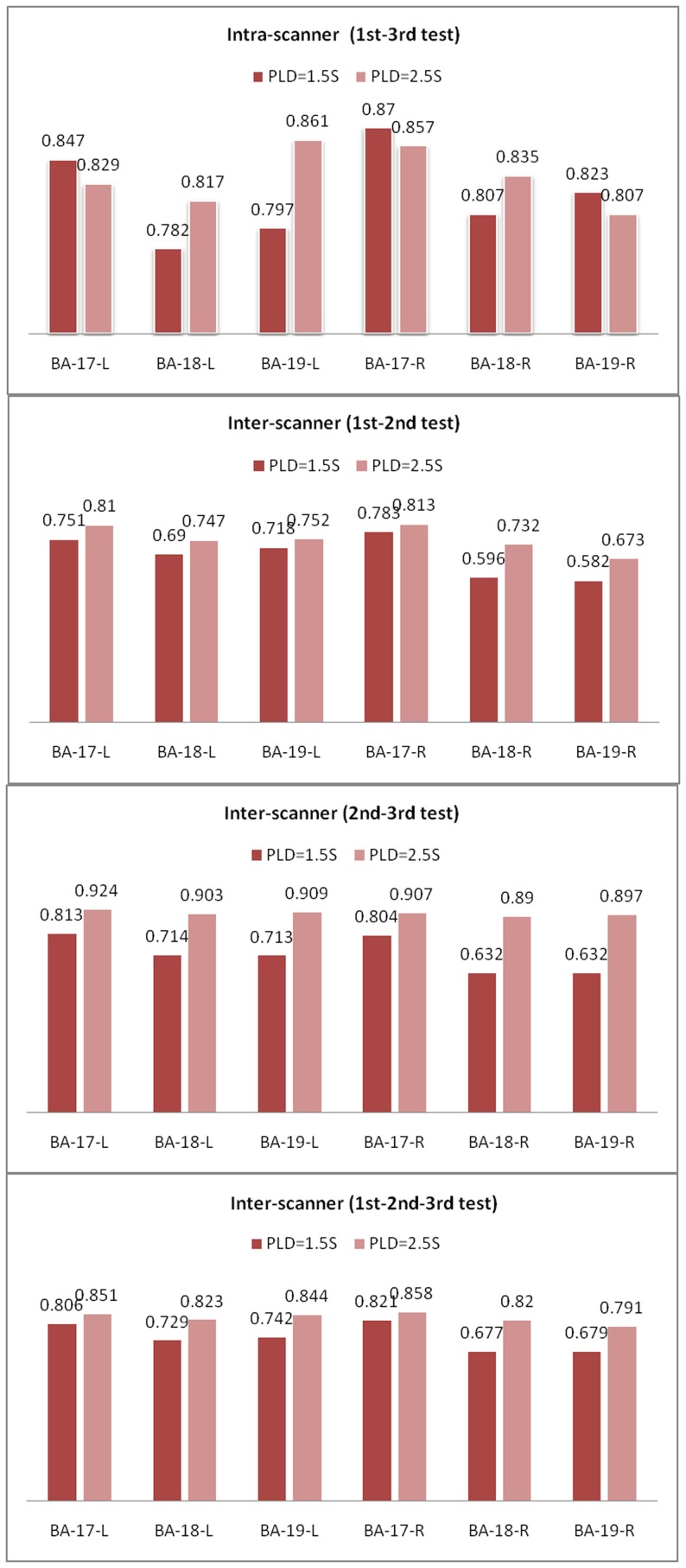
Corresponding ICC values of the six ROIs. Corresponding ICC values of the twelve volunteers for 6 ROIs for the 3D pCASL tests by two MR scanners using different PLDs. The ICC values in most of the ROIs (except for BA17 left and right, BA 19 right) were higher with PLD = 2.5 s than with PLD = 1.5 s. However, the selection of the PLD should take the ROI into consideration.

The representative ROIs of the twelve subjects are shown in Fig. 1. The summary of the average CBF and the SD of the BA 17, 18 and 19 ROIs are shown in Tables 1. The ICC values and 95% Confidence Interval (95%CIs) of the ROIs for the intra- and inter-scanner were shown in table 2. The correlation plots of the CBF values for all ROIs from the three tests using the two scanners are shown in Fig. 2, including the trend lines. The Bland–Altman plots together with the 95% CIs of the CBF measurements from all ROIs in the twelve volunteers are shown in Fig. 3. The linked-four histogram that determined the ICC values of the respective ROIs and the corresponding values are presented in Fig. 4. The separated ROIs are shown in Table 3 which suggested the resemblance of the bilateral visual cortex. Table 4 was added for revealing the mean CBF values for scan-1, scan-2, scan-3 which indicated higher mean CBF at the longer PLDs. Regarding the intra- and inter- scanner tests, the retests for the 3D pCASL displayed good correlations for most of the ROIs. As the PLD increased, the correlations became visibly better, and the distribution of the range of points became narrower. The mean CBF values of all the ROIs with PLD = 2.5 s were higher than those with PLD = 1.5 s, and the margins were small.

**Table 1:** The CBF values (mean and standard deviation) of the regions of interest (ROIs) for the pseudo-continuous arterial spin labeling (pCASL) imaging.

ROI name	CBF(PLD = 1.5 s)		CBF(PLD = 2.5 s)		D-value
	Mean	SD	Mean	SD	
Brodmann area 17-L	42.0957	9.8858	47.2688	9.4964	5.17
Brodmann area 17-R	46.8045	14.3077	51.3806	12.8383	4.58
Brodmann area 18-L	40.7709	13.3186	46.7427	10.8087	5.97
Brodmann area 18-R	42.7029	16.9757	47.9803	13.7285	5.28
Brodmann area 19-L	41.3857	11.447	48.5685	10.2792	7.18
Brodmann area 19-R	39.9187	13.5527	46.5859	11.5976	6.67

D-value: difference value, CBF: cerebral blood flow, PLD: post-labeling delay time.

**Table 2:** The ICC values and 95% Confidence Interval of the regions of interest (ROIs) for the intra- and inter-scanner the three-dimensional pseudo-continuous arterial spin labeling (3DpCASL) imaging.

PLD	Intra-scanner (1st–3rd test)	Inter-scanner (1st–2nd test)	Inter-scanner (2nd–3rd test)	Inter-scanner (1st–2nd–3rd test)
1.5 s	0.821	0.685	0.719	0.743
	0.728–0.884	0.540–0.790	0.586–0.814	0.649–0.821
2.5 s	0.831	0.754	0.903	0.829
	0.743–0.890	0.635–0.839	0.850–0.938	0.760–0.883

**Table 3:** The separated ICC values of the 6 ROIs for the intra- and inter-scanner using 3DpCASL.

ROI name	Intra-scanner (1st–3rd test)		Inter-scanner (1st–2nd test)		Inter-scanner (2nd–3rd test)		Inter-scanner (1st–2nd–3rd test)	
	PLD = 1.5 S	PLD = 2.5 S	PLD = 1.5 S	PLD = 2.5 S	PLD = 1.5 S	PLD = 2.5 S	PLD = 1.5 S	PLD = 2.5 S
Brodmann area 17-L	0.847	0.829	0.751	0.810	0.813	0.924	0.806	0.851
Brodmann area 17-R	0.782	0.817	0.690	0.747	0.714	0.903	0.729	0.823
Brodmann area 18-L	0.797	0.861	0.718	0.752	0.713	0.909	0.742	0.844
Brodmann area 18-R	0.870	0.857	0.783	0.813	0.804	0.907	0.821	0.858
Brodmann area 19-L	0.807	0.835	0.596	0.732	0.632	0.890	0.677	0.820
Brodmann area 19-R	0.823	0.807	0.582	0.673	0.632	0.897	0.679	0.791

**Table 4:** The mean CBF values for scan-1, scan-2, scan-3 using 3DpCASL.

SCAN NUMBER	Mean CBF value(PLD = 1.5 S)	Mean CBF value(PLD = 2.5 S)
Scan-1	40.7218	46.1994
Scan-2	43.0659	48.9518
Scan-3	43.0514	49.1122

The ICC values corresponding to the total ROIs were also calculated based on the mean CBF from all ROIs from each subject. The 3D pCASL consistently had high intra- and inter-scanner measurement reliability and reproducibility (i.e., the ICC of the total ROIs at PLD = 2.5 s is 0.829 compared to 0.743 at PLD = 1.5 s). The CBF reliability and reproducibility values showed homogeneity for all ROIs, which suggested the stability of the results.

**Figure 5:**
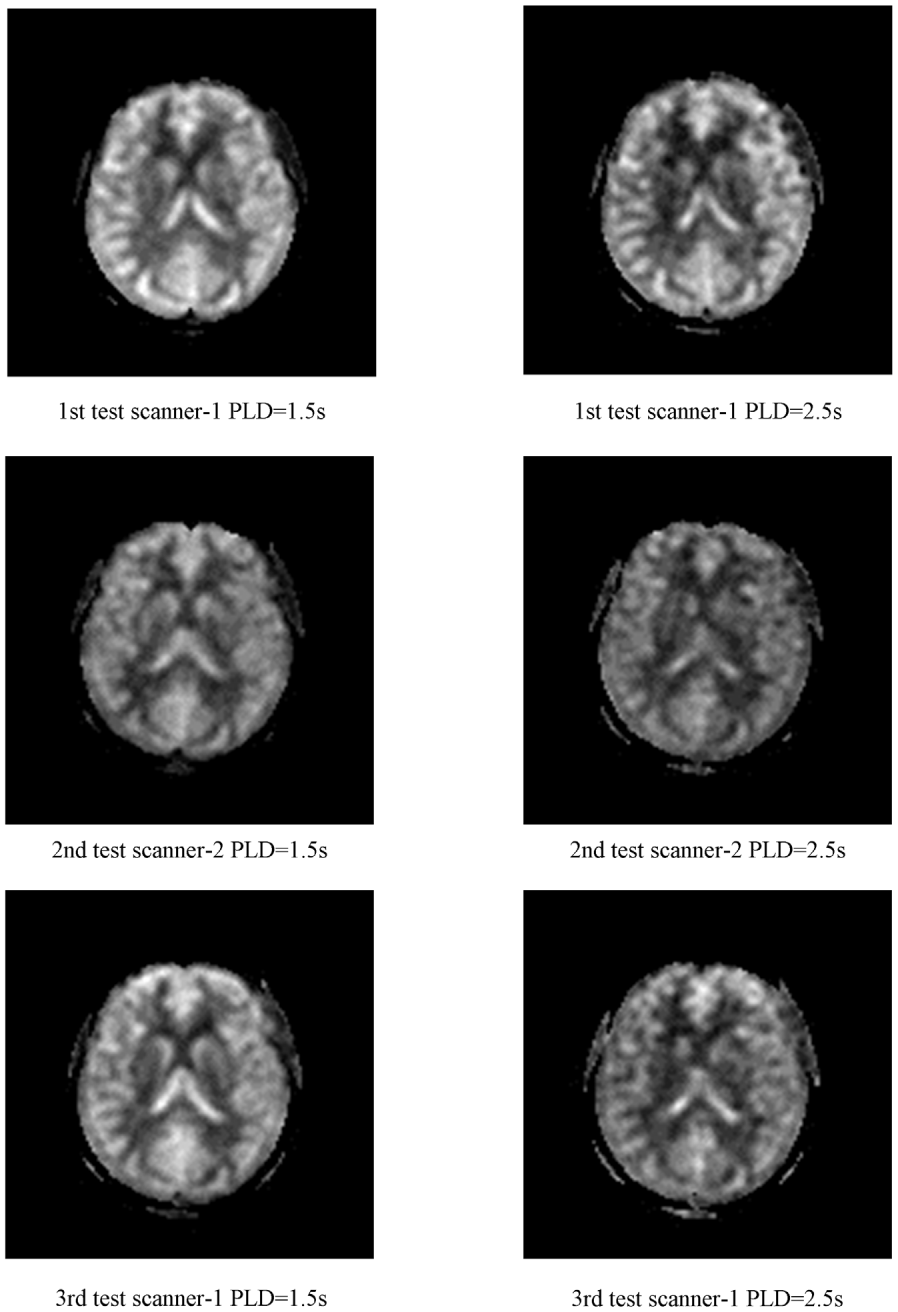
The perfusion maps of the different scanners with different PLDs. The perfusion maps of the different scanners with different PLDs of one subject were shown.

The mean number of days in the scan sessions was thirteen days. And the SNR at the PLD = 1.5 s is 21.83 while is 11.74 at PLD = 2.5 s which revealed the decrease of the SNR at longer PLDs. Besides, the perfusion maps of the different scanners with different PLDs of one subject were shown on Fig. 5.

There are statistical significance between the left and right BA 17 and 18(P<0.05), while there are no statistical significance in the BA19 left and right (P = 0.17>0.05)

## Discussion

The human visual cortex is partitioned into a number of functional areas with specific local neuronal properties [25], [26], [27], whereas with respect to its anatomy, it is mainly divided into the primary visual cortex (presented by BA 17) and the secondary visual cortex (presented by BA 18 and BA 19), which participates in the recognition of objects (shape and color) by the visual pathway. Alterations in the small blood vessels and perivascular cells can result in an inability to maintain regional cerebral blood flow and thus predispose these regions to ischemia/reperfusion injury [28]. By detecting the CBF in the visual cortex, we can predict mild changes in the visual cortex for the pathogenesis of some posterior visual pathway diseases.

In this study, the 3D pCASL technique was used to detect the CBF of the visual cortex, which is rarely affected by remote draining veins. Therefore, 3D pCASL, which is spatially and temporally more closely linked to the underlying CBF of the visual cortex, can be used to determine the fluctuation in the CBF in the posterior visual pathway.

The ICC values were calculated in the current research using ROIs to assess the reliability and reproducibility of different regional CBFs in the visual cortex. The results revealed that the 3D pCASL had high intra- and inter-scanner measurement reliability (i.e., the ICC of the total ROIs were greater than 0.829 and 0.743, respectively). Additionally, the fluctuation of all the ROIs showed stable ICC values, ranging from 0.582 (BA 19 right, PLD = 1.5 s) to 0.924 (BA 17 left, PLD = 2.5 s). These findings showed a more stable reliability compared with the previous study [29]. There are two relatively novel findings going to explore in the following. On one side, the ICC for the longer PLD resulted in a higher value than the shorter PLD for the total ROIs. Such results implied that the heterogeneity of the PLD times between subjects and among different visual regions affected the reliability and reproducibility of the flow quantification. However, the conclusion should be prudently applied to the detection of posterior disease with ASL due to the selection of the PLD in different ROIs implied diverse ICC values and only few studies used the PLD selected in the visual cortex. The magnetically labeled water in ASL only has a half-life of 1–3 seconds compared with the 2 minutes of half-life for 15O-water. Therefore, with multiple PLDs, the 3D pCASL technology may better explore the CBF of the visual cortex for clinical applications. On the other side, the longer PLD presents not only higher ICC values but also the higher perfusion values. PLD refers to the time at which the end of the labeled bolus leaves the labeling plane in pCASL. It should be understood that areas of low perfusion may reflect some combination of low CBF and unusually long arterial transit time (ATT), and not specifically low CBF. The borderzone or watershed areas such as BA17, 18 and 19 are at the relative distal portions of each vascular territory, and will naturally have a longer ATT than other portions of the territory. Note that it is possible for low ASL signal in these regions to represent long ATT rather than low CBF, and an additional scan with longer PLD may help to distinguish between these two possibilities. According to all the above, further ATT study should be considered.

The primary visual cortex (BA 17) presented an obviously more stable and higher ICC value than the BA 18 and 19, which suggested that the primary visual cortex may be less affected by external interference. These findings may occur because BA 17 rarely participates in occipital dysfunction whereas the secondary visual pathway, mainly including BA 18 and 19, can induce dysfunction in the recognition of an object's shape and color, leading to visual misidentifications such as visual hallucinations [30]. Furthermore, this finding may suggest that BA 17 occupies a more important status in the visual apperception and visual transduction pathway, which requires a more stable CBF to be protected. The relationship between the CBF value of the striate and peristriate cortices and neuro-ophthalmological diseases needs further study.

The comparison between the left and right BA 17 and 18 showed significant difference (P<0.05), while there are no statistical significance in the BA19 left and right (P = 0.17>0.05). The difference CBF between the left and right in the visual cortex may relate to the dominant eye and the correlation between visual acuity and the ventrally located visual areas which need further study. Based on the discovery above, 3D pCASL could be used in research performed at multiple centers and in long-term observations of clinical effects.

Some of the limitations of the present study should be recognized. First, the ASL data from patients could not be included. Because cerebral perfusion might be lower in patients with neuro-ophthalmological diseases than in healthy subjects, we may have overestimated the reliability of ASL in actual patients. The advantages of excluding patients included the possibility of reduced head movements and decreased disease-related variability. Second, the CBF value was not entirely stable while the unstability was in control in healthy subject. However, for some research questions, this limitation may be outweighed by the 3D pCASL technique's ability to measure the activation associated with longitudinal changes in performance during learning or blood flow recovery and its ability to distinguish the direct cardiovascular effects of psychoactive drugs from secondary effects related to changes in processing. Third, the SNR at the PLD = 1.5 s is 21.83 while is 11.74 at PLD = 2.5 s which verified the decrease of SNR with longer PLD. However, to avoid the influence on the result and improve perfusion SNR, the current pCASL technique adapted a combination of several technical advances, including a 3.0T scanner, a multichannel receiver coil, background static signal suppression, and 3D spiral FSE acquisition. Finally, all patients kept their eyes closed in our study to eliminate any effects of fMRI activity in the visual cortex [31]. Because the relationship between the CBF with eyes open and closed remains unknown, it requires further study.

In conclusion, the reproducibility of the measurements of 3D pCASL inter- and intra-scanners across a time interval was determined within the visual cortex for ROIs based on CBF activation. Although the inter-scanner reliability was slightly lower than the intra-scanner reliability, these outcomes across different times and with two scanners were similar. Our study demonstrated that the 3D pCASL can be used to conduct research in multiple centers, for the long-term observation of neuro-ophthalmological clinical effects and to study the pathology and diagnosis of posterior visual pathway diseases with appropriately selected PLDs.
